# Influenza-induced immune suppression to methicillin-resistant *Staphylococcus aureus* is mediated by TLR9

**DOI:** 10.1371/journal.ppat.1007560

**Published:** 2019-01-25

**Authors:** Giovanny J. Martínez-Colón, Helen Warheit-Niemi, Stephen J. Gurczynski, Quincy M. Taylor, Carol A. Wilke, Amy B. Podsiad, Joel Crespo, Urvashi Bhan, Bethany B. Moore

**Affiliations:** 1 Graduate Program in Immunology, University of Michigan, Ann Arbor, MI, United States of America; 2 Microbiology and Immunology Graduate Program, University of Michigan, Ann Arbor, MI United States of America; 3 Pulmonary and Critical Care Medicine Division, Department of Internal Medicine, University of Michigan, Ann Arbor, MI, United States of America; 4 Literature, Sciences and the Arts, Microbiology, University of Michigan, Ann Arbor, MI, United States of America; 5 Department of Microbiology and Immunology, University of Michigan, Ann Arbor, MI, United States of America; University of Minnesota, UNITED STATES

## Abstract

Bacterial lung infections, particularly with methicillin-resistant *Staphylococcus aureus* (MRSA), increase mortality following influenza infection, but the mechanisms remain unclear. Here we show that expression of TLR9, a microbial DNA sensor, is increased in murine lung macrophages, dendritic cells, CD8^+^ T cells and epithelial cells post-influenza infection. TLR9^-/-^ mice did not show differences in handling influenza nor MRSA infection alone. However, TLR9^-/-^ mice have improved survival and bacterial clearance in the lung post-influenza and MRSA dual infection, with no difference in viral load during dual infection. We demonstrate that TLR9 is upregulated on macrophages even when they are not themselves infected, suggesting that TLR9 upregulation is related to soluble mediators. We rule out a role for elevations in interferon-γ (IFNγ) in mediating the beneficial MRSA clearance in TLR9^-/-^ mice. While macrophages from WT and TLR9^-/-^ mice show similar phagocytosis and bacterial killing to MRSA alone, following influenza infection, there is a marked upregulation of scavenger receptor A and MRSA phagocytosis as well as inducible nitric oxide synthase (Inos) and improved bacterial killing that is specific to TLR9-deficient cells. Bone marrow transplant chimera experiments and in vitro experiments using TLR9 antagonists suggest TLR9 expression on non-hematopoietic cells, rather than the macrophages themselves, is important for regulating myeloid cell function. Interestingly, improved bacterial clearance post-dual infection was restricted to MRSA, as there was no difference in the clearance of *Streptococcus pneumoniae*. Taken together these data show a surprising inhibitory role for TLR9 signaling in mediating clearance of MRSA that manifests following influenza infection.

## Author summary

Influenza-associated secondary bacterial infections, particularly with methicillin-resistant *Staphylococcus aureus* (MRSA), are a major cause of morbidity and mortality, and better therapeutic strategies are needed. Stimulation of TLR2 has shown promise for improving health in influenza-bacteria dual-infected animals. However, nothing is known about the role of other TLRs, including TLR9, in influenza-bacteria dual infection pathology. This is the first study of TLR9 regulation of influenza-bacterial superinfection and it highlights an unexpected pathologic role for TLR9 in regulating clearance of MRSA post-H1N1. It also highlights the important observation that TLR9 signaling has very different outcomes in the setting of influenza infection than in naïve mice and shows important distinctions in the mechanisms for susceptibility to MRSA vs. *S*. *pneumoniae* post-influenza. Our results also suggest that TLR9 expression on non-hematopoietic cells regulates macrophage function in vivo.

## Introduction

Influenza viruses are single-stranded RNA viruses with a segmented genome capable of undergoing mutagenesis to evade host immunity and they cause seasonal outbreaks leading to over a half million deaths per year worldwide (World Health Organization, 2016)[1]. Influenza viruses can overcome traditional vaccine strategies such as inoculation with inactivated viruses, as this will not confer long-lasting protection to antigenic drift [2]. There are three types of influenza viruses that can infect humans (A, B, and C). Influenza A virus (IAV) and influenza B can both cause seasonal outbreaks but IAV is generally more severe. IAV infections can be complicated by bacterial pathogens including *Staphylococcus aureus*, and *Streptococcus pneumoniae* leading to increased morbidity and mortality [3]. Retrospective studies have shown that 95% of the deaths caused by the 1918 influenza pandemic (Spanish Flu) were complicated by bacterial superinfections [3, 4]. More recently, whole-blood transcriptome analysis of over 225 influenza-infected patients showed a shift and enrichment in gene signatures from viral response to bacterial response in critically ill patients [5]. Thus, to decrease morbidity and mortality of IAV infections we need a better understanding of how to treat secondary bacterial infections. Past studies have found that IAV infections can lead to secondary bacterial infections by increasing the attachment sites for bacteria, reducing responsiveness of immune cells, and reducing efficiency of antibiotics [6–8]. Yet, the mechanisms underlying influenza-induced mortality are still poorly understood and we need better therapeutic strategies to improve outcomes for influenza-infected individuals.

Toll-like receptors (TLRs) are germline encoded pathogen recognition receptors (PRRs) capable of initiating innate immune responses, and regulating adaptive immunity to both viral and bacterial pathogens [9]. They are primarily expressed in immune cells, and are membrane bound and distributed in the extracellular membrane and endosomes making them able to recognize extracellular and intracellular pathogen components [10]. Manipulation of TLRs has shown great potential in combating bacterial infections. For example, agonistic stimulation of TLR4, a lipopolysaccharide (LPS) receptor, has been shown to improve bacterial clearance in *Pseudomonas aeruginosa* infected mice [11]. IAV has been shown to alter the expression of TLRs including downregulation of TLR2, a bacterial lipopeptide sensor, in human monocytes and dendritic cells [12]. TLR2 agonist stimulation was shown to have therapeutic potential to improve survival as well as bacterial and viral clearance in a mouse model of viral-bacterial coinfection [13]. However, little is known about the role of other TLRs that are altered in IAV infections and their implications in secondary bacterial infections.

IAV infection was shown to increase the expression of TLR9 in human monocytes and dendritic cells [12]. TLR9 is an endosomal receptor that recognizes unmethylated cytosine and guanine (CpG) motifs which are rich in viral and bacterial DNA and mitochondrial DNA (mtDNA)[14, 15]. Here, we aimed to study the role of TLR9 in IAV-associated bacterial secondary infections, particularly with the bacterial pathogen methicillin-resistant *Staphylococcus aureus* (MRSA). Studying MRSA secondary infections is of high importance as in recent pandemics it was the main cause of secondary pneumonia in IAV infected individuals [16]. Additionally, MRSA is the leading cause of bacterial infections in humans worldwide, and infections are difficult to treat as MRSA is resistant to all known β-lactam antibiotics [17].

With the use of a mouse-adapted IAV strain, A/Puerto Rico/8/1934 (PR8), we found that TLR9 expression is elevated in lung macrophages, dendritic cells, CD8 T cells and epithelial cells from PR8 infected mice. TLR9^-/-^ mice infected with PR8 or MRSA alone did not differ in clearance of either pathogen from wild-type (WT) mice, but they experience improved survival post PR8-MRSA dual infection and show improved bacterial phagocytosis and killing post dual infection Our findings show a previously unrecognized role for TLR9 in limiting clearance of MRSA post-dual infection.

## Results

### IAV increases expression of TLR9 in macrophages

**Fig 1:**
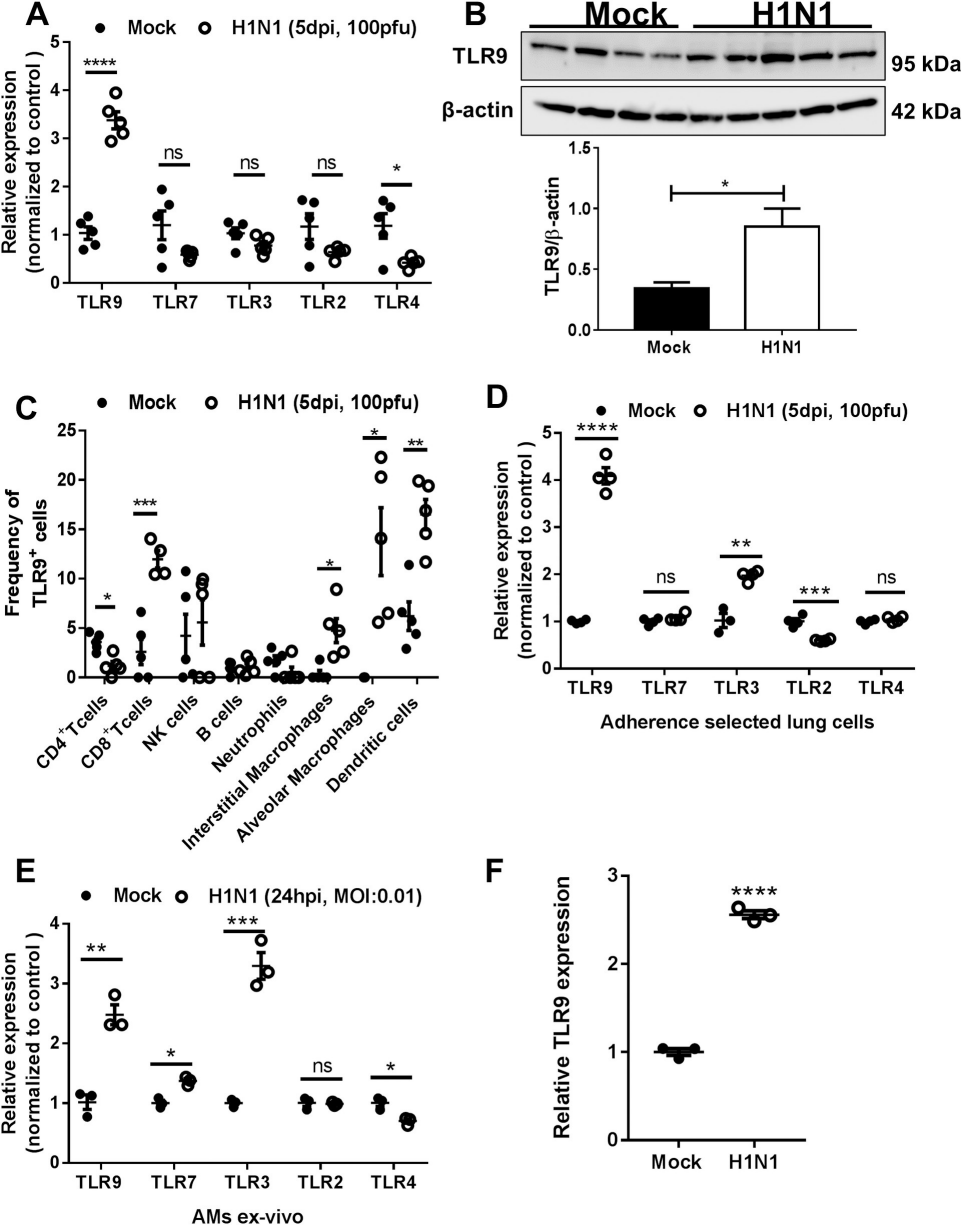
TLR9 overexpression in lung immune cells post-IAV infection. (A) Relative gene expression of TLRs (9, 7, 3, 2, 4) by RTqPCR and (B) western blotting of TLR9 and β-actin. RNA and protein were isolated from lung leukocytes post-collagenase digestion in mice infected with 100 PFUs of H1N1 (PR8) for 5 days or placebo (PBS). β-actin was used to normalize RNA in samples. (C) Frequency of TLR9^+^ cells measured by flow cytometry in lung immune cells post-collagenase digestion. Gating was as follows: CD4^+^ T cells (CD45^+^,CD90.2^+^, CD3^+^, CD4^+^), CD8^+^ T cells (CD45^+^,CD90.2^+^, CD3^+^, CD4^-^, CD8^+^), natural killer cells (CD45^+^,CD90.2^+,^ NKp46^+^), B cells (CD45^+^,CD90.2^-^, CD19^+^), neutrophils (CD45^+^, CD11b^+^, LY6G^+^), interstitial macrophages (CD45^+^, CD64^+^, CD11b^+^, F4/80^+^), alveolar macrophages (CD45^+^, CD64^+^, CD11C^+^, Siglec F^+^) and dendritic cells (CD45^+^, CD64^-^, CD11c^+^, MCHII^high^). Staining for TLR9 was performed on cells which were fixed and permeabilized. (D) Relative expression of TLRs (9, 7, 3, 2, 4) after lung macrophage isolation by adherence selection from PBS or IAV-infected mice. (E) Relative expression of TLRs (9, 7, 3, 2, 4) in alveolar macrophages infected or not ex-vivo with H1N1 (MOI:0.01) for 24 hours. (F) BMDMs from Balb/c mice infected with H1N1 at MOI:0.01 and measured for TLR9 mRNA after 48h. Statistics are student T test between comparative groups. *P<0.05,**P<0.01, ***P<0.001, ****P<0.0001. Experiments in panels A, D, E and F were repeated at least 2 times with similar results. Panels B and C are single experiments on n = 4 (panel B) or n = 5 (panel C) mice.

Changes in expression of different toll-like receptors (TLRs) (TLR2, TLR3, TLR4, TLR7, TLR8, and TLR9) have been reported before in human monocytes and dendritic cells from seasonal influenza infected patients [12]. Similarly, in our murine experiments, we noted that TLR9 gene expression is increased in lung leukocytes obtained by collagenase digestion and ficoll density separation 5 days post-PR8 infection while TLR4 is reduced (Fig 1A). Protein expression was also increased in these lung leukocytes post-PR8 infection as measured by TLR9 immunoblotting (Fig 1B). To understand which cells were upregulating TLR9, we used flow cytometry to characterize the major immune cells in the lung compartment. We found that CD8 T cells, macrophages (interstitial and alveolar), and dendritic cells were the main cells with higher TLR9 expression post-PR8 (Fig 1C). We did not find changes in NK cells, B cells, or neutrophils but CD4 T cells showed a lower frequency of TLR9+ cells (Fig 1C). Adherence selection of lung leukocytes for 1 h after collagenase digestion enriches for myeloid cells such as monocytes and macrophages and allowed us to detect gene expression changes in multiple TLRs after influenza infection in these cells. We found that apart from TLR9 being increased, TLR3 and TLR2 were also altered with increased and decreased expression, respectively (Fig 1D). These later observations were also seen previously in human monocytes from influenza-infected individuals [12]. Interestingly, direct infection of isolated alveolar macrophages with PR8 also shows an increase in TLR9, TLR7 and TLR3, with no changes in TLR2, and reduced TLR4 (Fig 1E). We also detected TLR9 mRNA increased 24 hours post-PR8 infection in cultured bone marrow derived macrophages (BMDMs) (Fig 1F).

**Fig 2:**
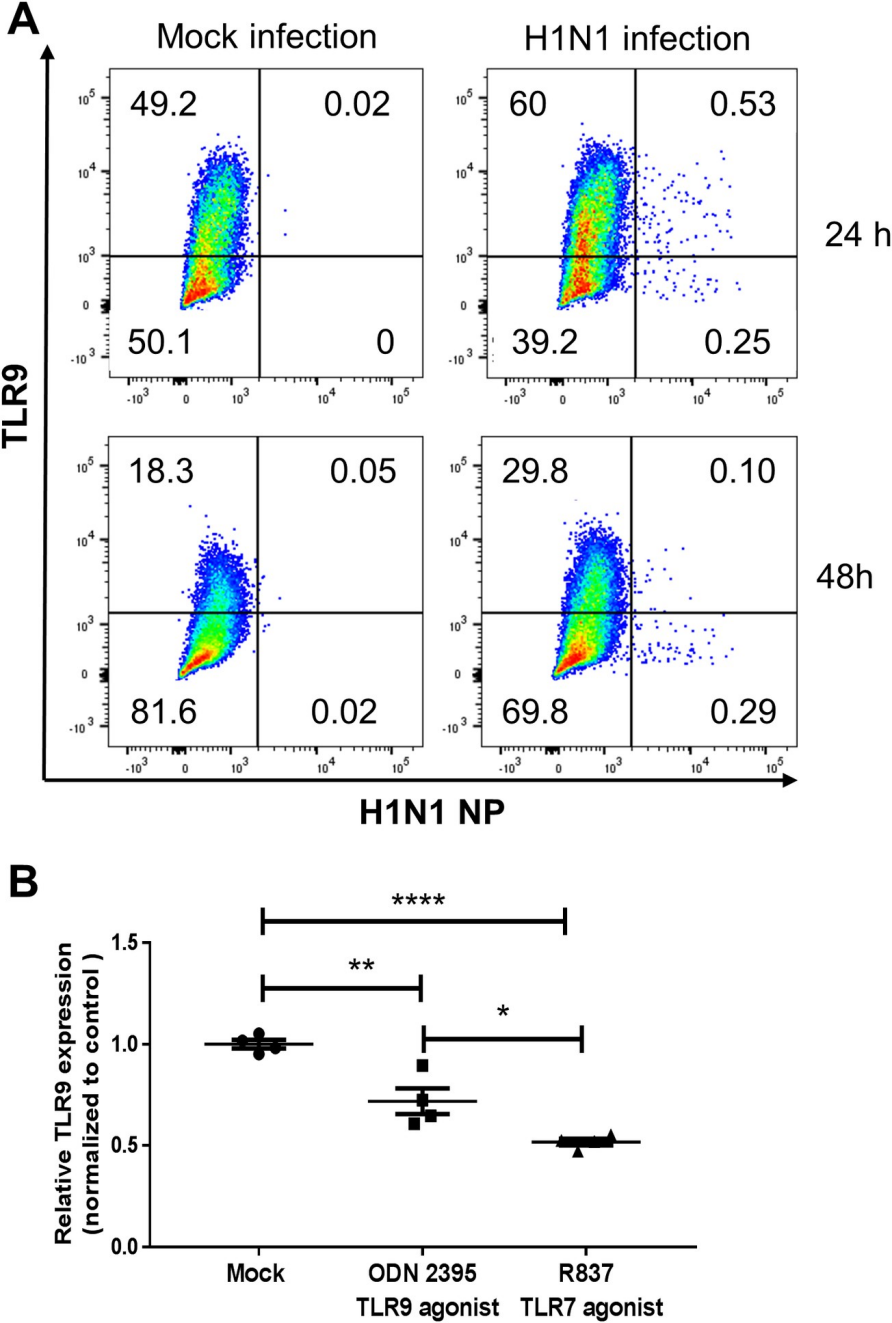
TLR9 is upregulated in non-infected BMDMs. (A) BMDMs were infected with MOI:0.01 H1N1 and cells were stained for expression of F480, TLR9 and NP by flow cytometry after 24 or 48 h. Flow plots are representative of n = 4–5 samples with similar results in 2 experiments. (B) BMDMs were treated with the TLR9 agonist, ODN 2395, or with the TLR7 agonist, imiquimod (R837), for 24 hours at a concentration of 1μM or 1μg/ml, respectively. RNA was isolated and TLR9 transcript expression was measured by RTqPCR; n = 4; Results analyzed by ANOVA with Tukey post-test.***P<0.001 and are indicative of two similar experiments.

In order to determine whether TLR9 was being upregulated only in infected macrophages or also in non-infected cells, we infected bone marrow derived macrophages (BMDMs) for 24 or 48h with H1N1 or mock infection. We then measured levels of H1N1 infection by expression of the viral protein, NP, and looked for TLR9 levels by flow cytometry on cells which also expressed CD45 and F4/80 (Fig 2A). TLR9 is expressed on 55.4 ± 1.6% of H1N1 infected BMDMs compared to 50.2 ± 1.3% of mock infected cells at 24 h whereas NP expression was noted in only 0.3 ± 0.07% of cells at this time point (n = 4–5 group, P<0.05 for mock vs. infected TLR9%, flow plots from one sample for each shown). At 48 h, expression of TLR9 in mock-infected samples was seen in 19.9 ± 1.0% of mock infected cells and 28.2 ±1.08% of H1N1 infected cells. By 48 h, 0.1 ± 0.008% of BMDMs were NP+ (n = 4–5 per group, P<0.001 for TLR9% between mock and H1N1-infected cells at 48 h). Furthermore, BMDMs were not productively infected by H1N1 as NP expression decreased from 24 to 48h. To our knowledge, we are the first to report that infection of macrophages ex vivo with IAV can increase TLR9 expression. The signal to mediate this increase was likely independent of IAV recognition by TLR7, or by IAV-induced release of CpG rich mitochondrial (mt)DNA as TLR7 and TLR9 agonist stimulation lead to downregulation of TLR9 gene expression relative to mock infection (Fig 2B). Taken together with the observation that TLR9 is increased on uninfected cells and on cell types not traditionally infected by IAV in vivo, these data are consistent with a secreted mediator being responsible for the upregulation of TLR9 expression post-H1N1 infection.

### TLR9^-/-^ mice show no difference in susceptibility to H1N1 or MRSA infection alone

**Fig 3:**
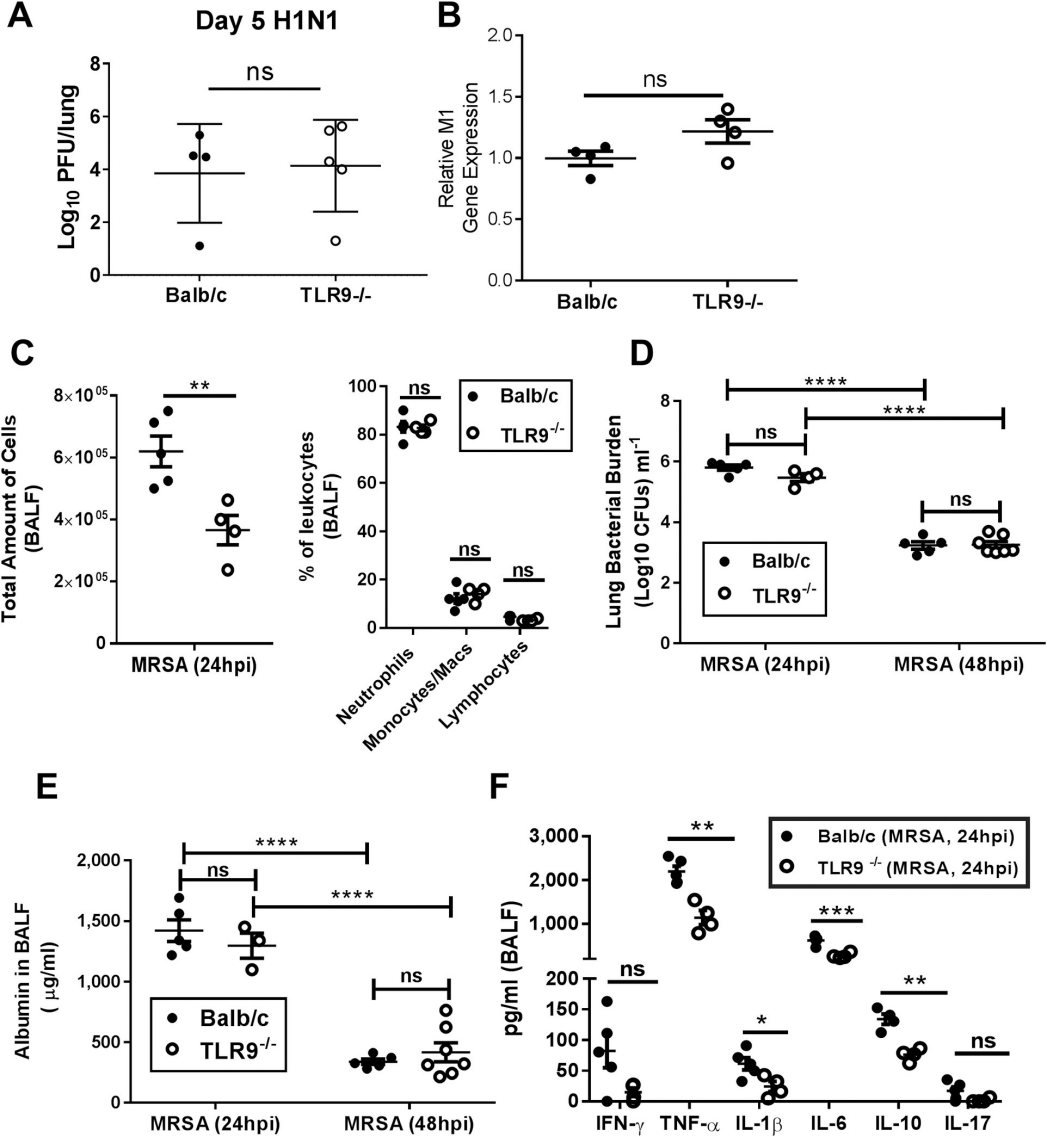
Balb/c and TLR9^-/-^ mice show similar susceptibility to H1N1 and MRSA alone. (A) Balb/c and TLR9^-/-^ mice were infected with 100 PFU H1N1 and lungs were collected for determination of viral load by plaque assay or (B) by detection of viral M1 gene levels; n = 4-5/group. Panel A was repeated 2 times and panel B is a single experiment done to confirm results of plaque assay. Student’s T-test shows results are not significantly different between genotypes. (C) Total number of cells (left) and percentage of leukocytes (right) in the alveolar compartment of BALB/c and TLR9^-/-^ mice infected with MRSA alone and quantified using a hemocytometer and differential staining; samples were taken by bronchoalveolar lavage 24 hours post-MRSA (7x10^7^ CFUs) infection. (D) Lung bacterial burden and (E) albumin measurements in the BALF 24 and 48 hours post-MRSA (7x10^7^ CFUs) infection in BALB/c and TLR9^-/-^ mice. (F) Cytokine levels in the BALF of BALB/c and TLR9^-/-^ mice infected with MRSA (7x10^7^ CFUs) for 24 hours. Cytokines were measured by ELISA. (C) & (F) Statistics are student T test; ns = non-significant, *P<0.05, **P<0.01, ***P<0.001. Panel C was repeated twice and panel F is a single experiment with n = 4–5 mice/group. (D) and (E) statistics were obtained by one-way analysis of variance with Tukey’s posttest. ****P<0.0001. The 24 h time point was repeated at least 2 times and the 48h time point was a single experiment.

The upregulation of TLR9 following H1N1 infection suggested the potential for cross-talk between viral infection and bacterial genome sensing. However, before addressing this, we wanted to first determine whether loss of TLR9 had any impact on host defense against infection with H1N1 alone or MRSA alone. Fig 3A demonstrates that 5 days post-infection with 100 PFU H1N1, WT (Balb/c) and TLR9^-/-^ mice showed equivalent viral loads in the lung by plaque assay and in a separate experiment that viral M1 gene expression in the lung at this time was similar between genotypes (Fig 3B).

Currently, there are conflicting results regarding the role of TLR9 in single MRSA infection [18, 19]. To understand if TLR9^-/-^ mice were susceptible to a single MRSA infection, we monitored mice for 7 days post-infection. We did not detect any deaths in either TLR9^-/-^ or WT mice but weight recovery during the MRSA infection was slower in TLR9^-/-^ mice (S1 Fig). We detected reduced immune cell infiltration in the alveolar compartment post-MRSA infection alone in TLR9^-/-^ mice (Fig 3C). However, this did not affect bacterial clearance (Fig 3D) or lung injury (Fig 3E) as both were reduced significantly 48 h post-infection in both genotypes. Previous work has shown that TLR9^-/-^ mice have reduced TNF-α in the bronchoalveolar lavage fluid (BALF) post-MRSA [19]. We detected lower amounts of TNF-α, IL-6, IL-1β and IL-10 in the BALF of MRSA-infected TLR9^-/-^ mice (Fig 3F). Yet, lower amounts of these cytokines did not have a negative effect on bacterial clearance, lung injury, or survival. Reduced immune cell infiltration and the lower cytokine profile might be explained by lower NF-κB activation post-MRSA infection as TLR9 is a sensor of bacterial DNA [20].

### TLR9^-/-^ mice are resistant to IAV and MRSA dual infection

**Fig 4:**
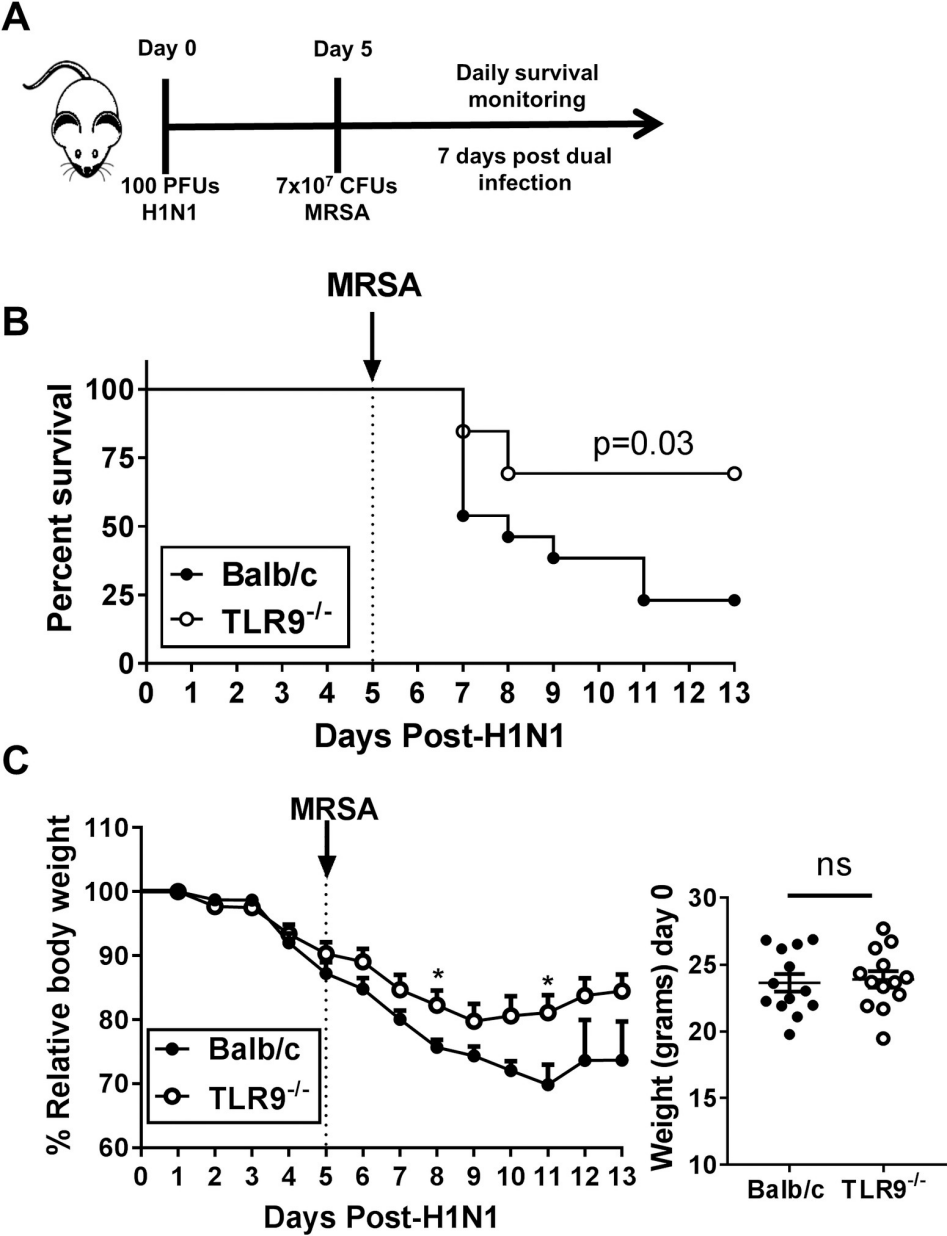
TLR9^-/-^ mice are resistant to secondary bacterial infection. (A) Sketch of infection model where Balb/c and TLR9^-/-^ mice are infected with 100 PFUs of H1N1 5 days prior to infection with 7x10^7^ CFUs of MRSA (US300). Mice are monitored daily to check for survival. (B) Survival assay of Balb/c (n = 13) and TLR9^-/-^ (n = 13) mice following coinfection. (C) Weight changes (left) and initial weight (right) of all mice used in survival assay. Data are combined from 2 independent experiments with mice matched for weight and sex at start. Statistics for weight changes on each day and initial weight were student T test, non-significant (ns); P<0.05 only on days 8 and 11. Statistics in survival assay were done with the Log-rank (Mantel-Cox), P = 0.03.

Secondary bacterial infections in IAV infected individuals are a main cause of mortality and morbidity [21]. To study the potential crosstalk between the virus and the bacteria, we tested IAV-MRSA coinfection models in Balb/c mice consisting of an initial viral infection (10 or 100 PFU) followed by a secondary bacterial infection on various days (S2 Fig). We determined that we could detect susceptibility to MRSA infection as early as 5 days post-100 PFU PR8 infection (S2B Fig; schematic diagrammed in Fig 4A). We next tested this dual infection model in WT and TLR9^-/-^ mice and observed a significant survival difference between TLR9^-/-^ and WT mice, where 77% of TLR9^-/-^ mice survived the secondary bacterial infection compared to 33% of WT mice (Fig 4B; data represent n = 13 mice combined from 2 separate survival assays matched for sex and starting weight). Surviving Balb/c mice on average weighed less than surviving TLR9^-/-^ mice but this only reached significance on days 8 and 11 (Fig 4C). In both experiments we noted that the TLR9^-/-^ mice appeared healthier than did the WT mice in terms of posture, grooming and activity in the cage post-dual infection.

### TLR9^-/-^ mice experience improved MRSA clearance post-dual infection

**Fig 5:**
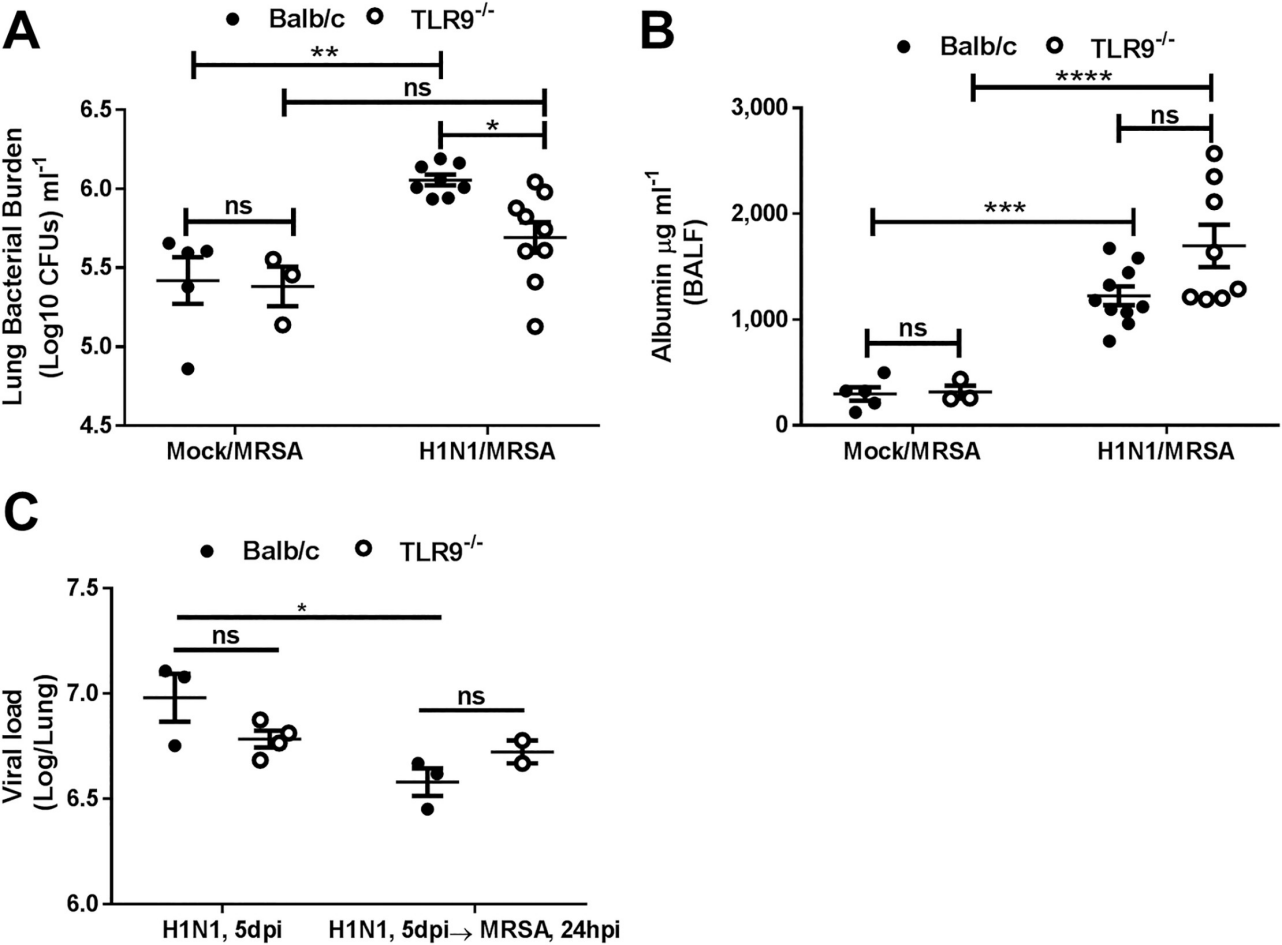
TLR9^-/-^ mice experience improved bacterial clearance post-IAV and MRSA coinfection. (A) Bacterial burden in lungs and (B) albumin levels in BALF from BALB/c and TLR9^-/-^ mice that were infected with IAV (100 PFUs, H1N1) or treated with placebo, PBS, 5 days prior to MRSA (7x10^7^ CFUs) infection; samples were harvested 24 hours post-MRSA infection. (C) Quantification of influenza titers in whole lung of BALB/c and TLR9^-/-^ mice infected with influenza (100 PFUs, H1N1) 5 days prior to MRSA (7x10^7^ CFUs) infection or PBS treatment. (A and C) Statistics were obtained by one-way analysis of variance with Bonferroni’s posttest, Panel B used Tukey’s post-test; ns = non-significant, *P<0.05, **P<0.01, ***P<0.001, ****P<0.0001. All experiments were repeated at least 2 times with similar results.

To determine whether the improved survival in the dual-infected TLR9^-/-^ mice corresponded with better bacterial clearance, WT and TLR9^-/-^ mice were infected with H1N1 on day 0 or were mock-infected. On day 5, mice received 7 x 10^7^ CFU MRSA and lungs were harvested 24h later. Fig 5A shows that in WT mice, preceding H1N1 infection impairs clearance of MRSA relative to mice getting mock infection prior to MRSA. Furthermore, we were able to detect better bacterial clearance in the IAV-infected TLR9^-/-^ mice 24 hours post-MRSA infection than in WT mice, but saw no difference in tissue injury (Fig 5B) or viral load (Fig 5C) in the presence or absence of MRSA between genotypes. Importantly, the difference we note in bacterial clearance at day 6 (24h post-MRSA infection) precedes the first deaths on day 7 (Fig 4B).

### Better bacterial clearance in TLR9^-/-^ mice post-dual infection is independent of increased levels of IFN-γ

**Fig 6:**
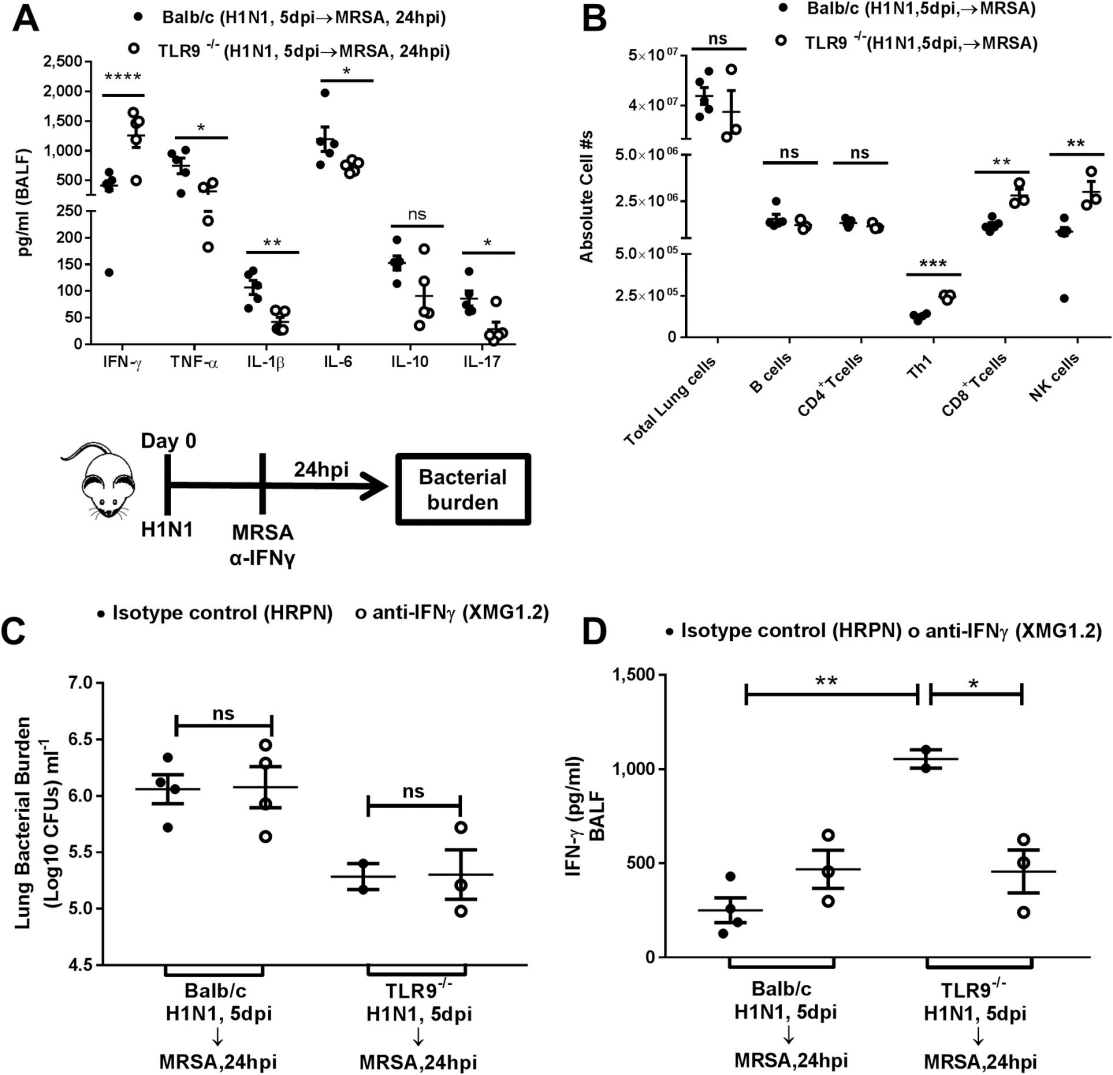
Higher bacterial clearance in TLR9^-/-^ mice post-IAV and MRSA coinfection is independent of exacerbated levels of IFN-γ. (A) Cytokine measurement in BALF from BALB/c and TLR9^-/-^ mice that were infected with IAV (100 PFUs, H1N1) 5 days prior to MRSA (7x10^7^ CFUs) infection. (B) Absolute number of lung immune cells post-lung collagenase digestion in BALB/c and TLR9^-/-^ mice that were infected with IAV (100 PFUs, H1N1) 5 days prior to MRSA (7x10^7^ CFUs) infection. Cells were quantified by flow cytometry; gating was as follow: B cells (CD45^+^CD90.2^-^CD19^+^); CD4^+^ T cells (CD45^+^CD90.2^+^CD4^+^); TH1 (CD45^+^CD90.2^+^CD4^+^,IFN-γ^+^); CD8^+^ T cells (CD45^+^CD90.2^+^CD4^-^,CD8^+^,IFN-γ^+^); NK cells (CD45^+^CD90.2^+^NKP46^+^,IFN-γ^+^). (C) Lung bacterial burden and (D) IFN-γ levels in BALB/c and TLR9^-/-^ mice that were treated with 200μg of IFN-γ neutralizing antibody or isotype control and infected with IAV (100 PFUs, H1N1) 5 days prior to MRSA (7x10^7^ CFUs) infection. (A-B) Statistics are student T test between comparative groups; ns = non-significant, *P<0.05, **P<0.01, ***P<0.001, ****P<0.0001. Panels A and B represent single experiments on 4–5 mice/group. (C-D) Statistics were obtained by one-way analysis of variance with Tukey’s posttest; ns = non-significant, *P<0.05, **P<0.01. Panels C and D represent a single experiment due to limited availability of neutralizing antibody.

To understand whether TLR9^-/-^ mice are clearing the bacteria better post dual infection due to differences in their cytokine profiles, we measured the levels of different pro and anti-inflammatory cytokines. We detected reduced amounts of TNF-α, IL-1β, IL-6, and IL-17, but increased levels of IFN-γ in TLR9^-/-^ mice (Fig 6A). High levels of IFN-γ have previously been shown to improve MRSA clearance due to full activation of macrophages [22]. Together with this, we detected higher numbers of TH1 (CD45^+^,CD90.2^+^,CD4^+^,IFN-γ^+^), CD8 T (CD45^+^,CD90.2^+^,CD4^-^,CD8^+^,IFN-γ^+^), and NK (CD45^+^,CD90.2^+^,NKP46^+^,IFN-γ^+^) cells, but no difference in other immune cells including B cells (CD45^+^,CD90.2^-^,CD19^+^), and CD4 T (CD45^+^,CD90.2^+^,CD4^+^) cells (Fig 6B). S3 Fig. shows the gating strategy for these analyses. To test whether high levels of IFN-γ in the bronchoalveolar lavage fluid (BALF) were responsible for the improved bacterial clearance in TLR9^-/-^ mice, we neutralized IFN-γ during IAV-MRSA coinfection. To our surprise, there was no difference between isotype-treated and IFN-γ-neutralized mice in terms of MRSA bacterial burden in either genotype (Fig 6C) even though we successfully neutralized the elevated IFN-γ levels in TLR9^-/-^ mice (Fig 6D).

### TLR9^-/-^ lung macrophages have increased phagocytosis, bacterial killing, and iNOS expression post-IAV infection

**Fig 7:**
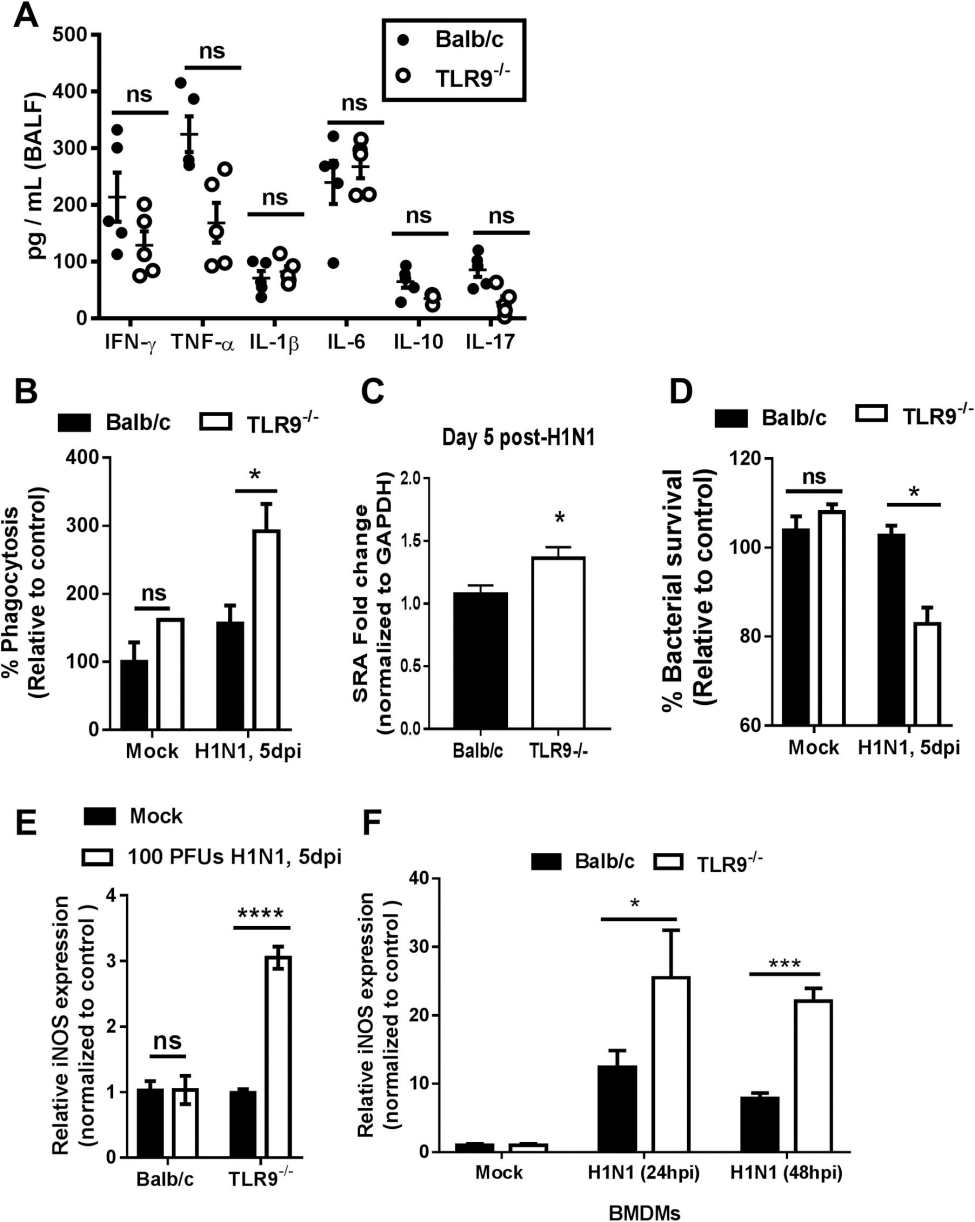
TLR9^-/-^ lung macrophages have increased phagocytosis, bacterial killing, and iNOS expression post-IAV infection. (A) Cytokine measurement in BALF from BALB/c and TLR9^-/-^ mice that were infected with IAV (100 PFUs, H1N1) for 5 days n = 5/group (some values are overlapping in the dot plots) for all but IL-10 which was from an experiment with n = 3 TLR9^-/-^ mice and 5 Balb/c mice. (B) Ex vivo MRSA phagocytosis by macrophages isolated by collagenase digestion and adherence purification from mock or H1N1 infected mice on day 5, n = 3. (C) SRA expression analyzed by real-time RT-PCR in monocyte/macrophages isolated on day 5 from H1N1-infected mice, n = 3. (D) Ex vivo MRSA killing assay using adherence selected lung macrophages from BALB/c and TLR9^-/-^ mice that were infected with IAV (100 PFUs H1N1) for 5 days or treated with PBS; n = 8/group in mock infections and n = 3/group in H1N1 infected mice. (E) Quantitative reverse transcriptase-PCR measurement of relative gene expression of iNOS from adherence selected lung macrophages from BALB/c and TLR9^-/-^ mice that were infected with IAV for 5 days or treated with PBS; RNA samples were normalized to their β-actin levels and setting mock-infected Balb/c to 1; n = 3-5/group. (F) BMDMs from Balb/c or TLR9-/- mice were mock-infected for 24 h or were infected with MOI:0.01 H1N1 for 24 or 48 h before RNA was prepared and analyzed for expression of iNOS normalized to their β-actin levels and setting mock-infected Balb/c to 1; n = 3/group. Panels A and C were analyzed by student’s t-test. Panels B, D, E and F were analyzed by two-way ANOVA with Sidak’s multiple comparison test.; Non-significant (ns), *P<0.05, ***P<0.001, ****P<0.0001. Panels A-D are single experiments using multiple mice, panels E and F were repeated two times.

To determine if IAV was inducing changes in TLR9^-/-^ mice that were leading to resistance to a MRSA infection, we measured the cytokine profile in the BALF in WT and TLR9^-/-^ mice post-single IAV infection. There were no differences in cytokines tested 5 days post-IAV infection (Fig 7A). We also found no significant differences in the immune cells recruited to the lungs (S4 Fig). Despite being present in equal numbers, monocyte/macrophages isolated from IAV-infected TLR9^-/-^ mice have increased MRSA phagocytosis (Fig 7B). This increased phagocytosis correlates with higher expression of scavenger receptor A (SRA) on monocyte/macrophages isolated on day 5 from TLR9^-/-^ versus WT mice infected with H1N1 (Fig 7C). Our laboratory has previously shown that phagocytosis of non-opsonized MRSA requires SRA expression [23]. TLR9^-/-^ mice also show improved intracellular killing of ingested bacteria (Fig 7D) and TLR9^-/-^ lung macrophages have higher levels of iNOS post-IAV compared to WT (Fig 7E). Nitric oxide production has been shown to be crucial in clearing MRSA infection [24]. To test whether TLR9 expression might suppress iNOS increase, we infected BMDMs from WT and TLR9^-/-^ mice and measured iNOS expression. TLR9^-/-^ macrophages have higher expression of iNOS post-IAV infection suggesting that TLR9 is a negative regulator of iNOS expression in BMDMs post-IAV infection (Fig 7F).

### Loss of TLR9 in hematopoietic cells or antagonism of TLR9 in macrophages is insufficient to improve MRSA clearance post-H1N1

**Fig 8:**
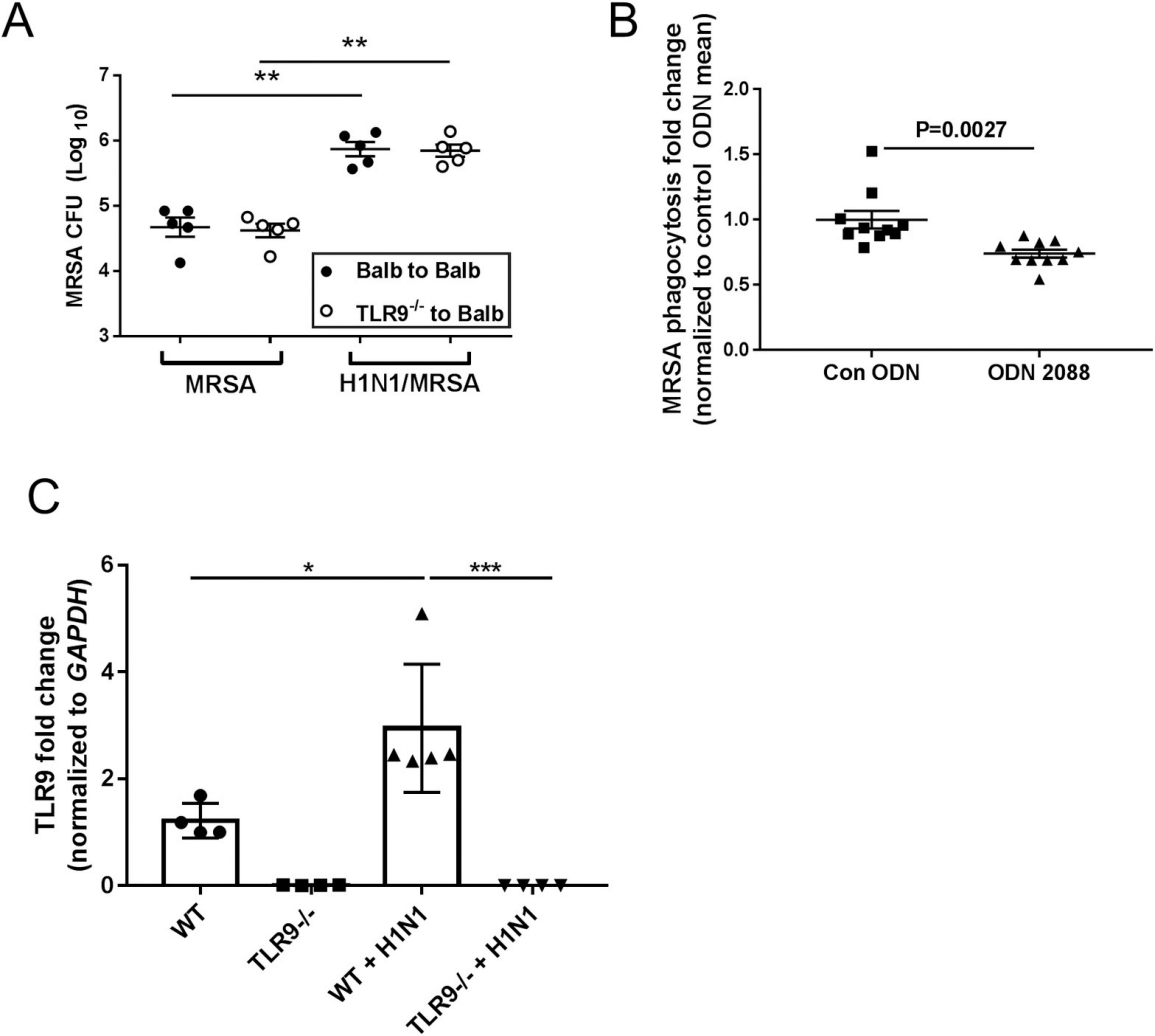
Improved clearance of MRSA post-H1N1 requires loss of TLR9 on non-hematopoietic cells; TLR9 is upregulated on lung epithelial cells post-infection. (A) Chimeric mice were generated by transplanting bone marrow from Balb/c or TLR9-/- mice into lethally irradiated Balb/c recipients. Following 5 weeks of engraftment, chimeric mice were infected with 7 x 10^7^ CFU MRSA alone or were infected with H1N1 (100 PFU) prior to infection with 7 x 10^7^ MRSA on day 5. Mice were harvested for CFU counts in lungs 24h post-MRSA infection. N = 5 mice/group, single experiment analyzed by ANOVA with Bonferroni post-test. **P<0.01. (B) Adherence purified monocytes and macrophages isolated on day 5 from H1N1-infected mice were treated with control ODN or the TLR9 antagonist ODN2088 at 10 μM concentration for 24h prior to infection with FITC-labeled MRSA for 2 h to measure phagocytosis. Single experiment with N = 10 replicates using cells combined from 3 mice. Data were analyzed by ANOVA with Tukey post-test. **P<0.01, ****P<0.0001. (C) Primary lung epithelial cells were isolated and infected ex vivo with MOI = 0.01 H1N1 or were mock infected for 24 h. Cells were then collected and analyzed for mRNA expression of TLR9. Symbols represent individual wells each with unique mock or H1N1 infection of cells pooled from 3 mice. Data were analyzed by ANOVA with Tukey post-test. *P<0.05, ***P<0.001.

To determine whether loss of TLR9 just in hematopoietic cells was needed for the beneficial effects on MRSA clearance, we created chimeric (WT into WT and TLR9^-/-^ in WT) mice and tested clearance of MRSA infection alone or clearance of MRSA following dual infection (Fig 8A). Surprisingly, loss of TLR9 in hematopoietic cells alone showed no benefit in clearance of MRSA alone or MRSA post-H1N1. Further proof that inhibition of TLR9 just in monocyte/macrophages was insufficient to improve MRSA clearance post-H1N1 is shown in Fig 8B where treatment of adherence purified monocytes and macrophages from H1N1-infeced mice with control oligodeoxynucleotide (ODN) or ODN2088, a TLR9 antagonist, demonstrated that ODN2088 treatment impaired (rather than improved) MRSA phagocytosis relative to control ODN-treated cells.

### Lung epithelial cells upregulate TLR9 post-H1N1

Taken together, the results in Fig 8A and 8B suggest that stimulation of TLR9 on structural or other non-hematopoietic cells of the lung likely causes release of soluble mediators that improve macrophage function in clearance of MRSA post-H1N1. To verify that TLR9 is modulated on lung structural cells post-H1N1 infection, we purified alveolar epithelial cells from WT or TLR9^-/-^ mice and infected them ex vivo with MOI = 0.01 H1N1 or cells were mock-infected. After 24 h, RNA was made from infected cells and analyzed for TLR9 gene expression. Fig 8C demonstrates that epithelial cells from WT mice upregulate TLR9 mRNA expression and confirm that TLR9^-/-^ mice do not express TLR9 transcripts.

## Discussion

Lower respiratory infections are the fourth leading cause of death with 3 million deaths each year worldwide (WHO, 2018). The influenza virus infects the upper and lower respiratory tract and is successful at infecting 3–5 million individuals each year, taking the life of nearly a half million of these individuals [1]. Severe illness and death in influenza infections are seen mostly in high risk subjects, the very young and the elderly. However, recent influenza outbreaks have taken the life of younger and healthier citizens creating public health concerns. Secondary bacterial superinfections are responsible for high morbidity and mortality in influenza-infected patients [21]. Even with proper care including influenza vaccines, hygiene, and antibiotics, influenza-associated secondary bacterial infections are a burden to public health [25]. Additionally, the over-use of antibiotics has led to the selection of multidrug resistant bacterial pathogens making it harder to reduce the severity of bacterial infections [17]. Thus, we are in need of better therapeutic strategies against viral-bacterial co-infections that can improve public health. Influenza infections have been shown to alter the expression of TLRs in immune cells [12]. Manipulation of TLRs, in particular TLR2, has been shown to improve survival, and microbial clearance in mice co-infected with influenza and bacterial pathogens [13]. However, little is known about the potential roles that other TLRs can have in controlling viral-bacterial co-infections.

TLR9 is an intracellular receptor that recognizes unmethylated CpG motifs which are rich in microbial DNA [14]. TLR9 expression was reported to be elevated in monocytes and dendritic cells from influenza-infected patients compared to healthy individuals [12]. Here, we noted that IAV infection similarly increases TLR9 expression in murine immune cells from mice infected with a mouse-adapted IAV strain (PR8) (Fig 1A–1D). This increase can also be achieved in cultured alveolar macrophages (Fig 1E) and BMDMs (Fig 1F) infected *in vitro*. We tested whether stimulation of TLR7, the innate influenza sensor could lead to increased expression of TLR9 as it is known that NF-κB activation by TLRs can induce TLR expression [26]. However, TLR7 stimulation actually reduced mRNA levels for TLR9 (Fig 2B). Influenza infections can lead to mitochondrial membrane permeabilization and release of mitochondrial components [27]. Release of mtDNA can also lead to activation of TLR9 due to mtDNA’s high concentration of unmethylated CpG [15, 20]. However, we found that CpG oligonucleotide stimulation of TLR9 also inhibited TLR9 mRNA (Fig 2B). Thus, it is still unclear how the IAV virus leads to the increased TLR9 expression noted in mice and humans. Because we see elevations of TLR9 in cells that are not actually infected with IAV, this mechanism is likely to be via secreted mediators and this will be a focus of our future investigations.

Mice lacking TLR9 (TLR9^-/-^ mice) did not differ in viral response against IAV compared to WT as there was no difference in measured viral titers or M1 viral gene expression (Fig 3A and 3B), cytokine profiles (Fig 6A), or immune cell infiltration (S4 Fig). However, TLR9^-/-^ mice were resistant to an IAV-MRSA coinfection with improved bacterial clearance (Fig 5A). The improved clearance of MRSA in TLR9^-/-^ mice was not due to a preexistent resistance to the bacteria as there was no difference in single MRSA infection between WT and TLR9^-/-^ mice (Fig 3D). Previous reports focused on the role of TLR9 in single MRSA infection have shown conflicting results. TLR9^-/-^ mice were reported to have decreased MRSA clearance despite showing a lower amount of TNF-α [19]. In contrast, MRSA was shown to induce a type I interferon response dependent on TLR9, and TLR9^-/-^ mice were reported to have lower TNF-α and improved bacterial clearance [18]. Similar to the previous findings, we noted a decrease in cytokine secretion in TLR9^-/-^ mice, specifically TNF-α, IL-6 and IL-10 were lower to single MRSA infection (Fig 3F); however, TLR9^-/-^ mice did not differ from WT in bacterial clearance, survival and tissue injury despite lower lung immune cell infiltration and cytokine release (Fig 3, S1 Fig). Thus, TLR9 seems to play a differential role in resistance to MRSA in the context of secondary bacterial infection post-IAV.

TLR9^-/-^ mice have increased IFN-γ in the BALF together with higher numbers of IFN-γ producing cells (TH1, CD8 T cells, and NK cells) in the lung post coinfection (Fig 6A and 6B). This increase in IFN-γ provided a potential explanation for the improved bacterial clearance in TLR9^-/-^ mice as IFN-γ has been shown to increase clearance of MRSA [22]. However, in our studies, INF-γ neutralization [confirmed by ELISA (Fig 6D)] was not able to decrease clearance of MRSA in TLR9^-/-^ mice (Fig 6C). Therefore, the enhanced clearance of MRSA is independent of IFN-γ. Previous findings have shown that *S*. *aureus* is able to evade immunity and survive inside cells including phagocytic cells [17]. This is consistent with our data showing that IFN-γ cannot improve killing of MRSA post-H1N1 (Fig 6C).

While elevated IFN-γ was not critical for MRSA clearance, previous studies have shown that IFN-γ plays a negative role in *Streptococcus pneumoniae* (SPS3) clearance post-IAV infection by decreasing the expression of macrophage receptor with collagenous structure (MARCO) [28]. Just like MRSA, SPS3 is a gram-positive bacterial pathogen that is a high threat to influenza-infected individuals [29, 30]. Thus, we wondered if TLR9^-/-^ mice would make higher levels of IFN-γ following dual infection with H1N1 + SPS3, and if so, if that would correlate with higher SPS3 bacterial loads. Interestingly, we found that TLR9^-/-^ mice have no difference in SPS3 clearance with or without an initial influenza infection (S5A Fig). Furthermore, IFN-γ levels in the BALF of TLR9^-/-^ mice after IAV-SPS3 infection were not significantly higher than in dual-infected WT mice (S5B Fig). These findings highlight the important observation that TLR9 signaling has very different outcomes in the setting of influenza infection than in naïve mice and shows important distinctions in the mechanisms for susceptibility to MRSA vs. *S*. *pneumoniae* post-influenza.

Shortly after infection, MRSA is engulfed by phagocytes [31]. Macrophages, especially M1 (antimicrobial) polarized macrophages, play an essential role in the clearance of MRSA [32]. So we tested the ability of macrophages from TLR9^-/-^ mice to clear MRSA in culture. TLR9^-/-^ macrophages isolated from mock-infected mice had no difference in phagocytosis or intracellular bacterial clearance compared to WT (Fig 7B and 7D). However, macrophages from IAV-infected TLR9^-/-^ mice are capable of improving bacterial clearance and killing (Fig 7B and 7D). The increased phagocytosis is likely related to the elevated SRA expression on macrophages from TLR9^-/-^ mice post-H1N1 (Fig 7C). Interestingly, TLR9^-/-^ monocyte/macrophages from infected mice have higher expression of iNOS (Fig 7E). Similarly, BMDMs from TLR9^-/-^ mice show higher iNOS expression following ex vivo infection with H1N1 (Fig 7F). It has previously been reported that mice lacking iNOS expression are deficient in clearance of MRSA and more than 50% of mice will not survive a MRSA infection past 24 hours [24]. Thus, induction of iNOS is a likely explanation for why TLR9^-/-^ macrophages are more effective at clearing MRSA post-IAV infection.

Our results suggest that neutrophil accumulation is similar between WT and TLR9^-/-^ mice in response to MRSA alone (Fig 3B) or following H1N1 infection (S4 Fig). In Fig 7 we show that lung monocyte/macrophages show improved phagocytosis and bacterial killing against MRSA in TLR9^-/-^ mice. These results suggested that hematopoietic innate immune cells are primarily responsible for MRSA clearance. To determine whether the effects of TLR9 inhibition were localized to the myeloid immune cells, we created bone marrow chimeras to explore outcomes in mice which lacked TLR9 solely in the hematopoietic compartment. Interestingly however, these chimeras (WT into WT and TLR9^-/-^ into WT) showed equivalent clearance of MRSA both alone and post-H1N1 (Fig 8A). This suggests that the beneficial effects of TLR loss may be due to non-hematopoietic cell signaling. In this regard, it is interesting that we have noted TLR9 upregulation on lung epithelial cells infected ex vivo with H1N1 (Fig 8C). We attempted to treat mice with 2088 ODN or control ODN to see if TLR9 antagonism in wild-type mice was beneficial. However, these results were variable at the highest dosage of ODN 2088 tested (50 μg given on days 0, 2 and 5 i.p.). We believe this reflects the fact that antagonism of structural or other non-hematopoietic cells is needed and thus our dosage may not have been optimal. Future experiments will explore WT into TLR9^-/-^ chimeric mice for outcomes and will also explore lung-specific delivery of the ODN 2088.

In conclusion, our findings provide evidence that TLR9 plays a negative role in IAV-associated secondary MRSA infections. Blocking of TLR9 post-IAV infection can improve MRSA clearance and TLR9^-/-^ monocytes/macrophages show increased bacterial phagocytosis and intracellular killing post-IAV infection. Taken together, this suggests that TLR9 antagonism may be an effective therapeutic for MRSA complicated influenza infections assuming proper dosing can be identified. However, care should be taken to know the nature of the secondary infection as TLR9 regulates MRSA, but not SPS3 coinfection. Future work will be focused on elucidating the mechanism(s) of influenza-induced upregulation of TLR9 and the pathways which TLR9 alters to regulate MRSA killing.

## Material and methods

### Mice

BALB/c mice were bred in the animal facilities at the University of Michigan (Ann Arbor, MI). Breeding colonies of TLR9^-/-^ mice, on a BALB/c background, were kindly donated by Dr. Shizuo Akira [14] and were also bred in the animal facilities of the University of Michigan (Ann Arbor, MI). All mice used were at least 6–7 weeks old by the time of infection and/or treatment.

### Chimeric BMT mice

Balb/c mice were treated with 9 Gy total body irradiation split dose and infused with 5 million whole bone marrow cells from either Balb/c or TLR9-/- mice. Chimeric mice were given acidified water (pH 3.3) for 3 weeks post-transplant and were used for experiments at 5 weeks post-BMT.

### Ethics statement

Experiments were approved by the University of Michigan Institutional Animal Care and Use Committee under protocol PRO 7857. The animal use procedures are in compliance with University guidelines, State and Federal regulations and the standards of the “Guide for the Care and Use of Laboratory Animals. The University’s Animal Welfare Assurance Number on file with the NIH Office of Laboratory Animal

Welfare (OLAW) is A3114-01, and our facilities are accredited by the Association for the

Assessment and Accreditation of Laboratory Animal Care International.

### Bacteria and virus

*Staphylococcus aureus* (US300) was grown in nutrient broth and incubated with gentle agitation overnight at 37°C. *Streptococcus pneumoniae* (SPS3) (serotype 3, 6303) was grown in Todd Hewitt Broth with 0.5% yeast extract and incubated overnight in anaerobic conditions at 37°C and 5% CO_2_. Colony forming units (CFUs) were determined by optical density relative to known standard curves. Influenza A virus (IAV) (H1N1) strain A/PR/8/34 (PR8) was purchased from ATCC.

### Cells

Alveolar Macrophages (AMs) were isolated by bronchoalveolar lavage performed with supplemented Dulbecco’s Modified Eagle Medium (DMEM) (89% DMEM, 10% fetal bovine serum (FBS), and 1% penicillin-streptomycin (Pen-Strep) mixture) containing 5mM of ethylenediaminetetraacetic acid (EDTA). Total lung leukocytes were obtained by perfusing the lung with PBS followed by digesting the whole lung with Collagenase A and DNAse followed by Ficoll density separation as we have described [33]. Lung monocyte/macrophages were selected from these lung leukocytes by adherence to tissue culture plastic for 1 h and then cells were washed twice with PBS. Over 90% of attached cells were monocyte/macrophages (myeloid cells) by differential staining of attached cells, with the remaining cells largely neutrophils. Bone marrow derived macrophages (BMDMs) were obtained by differentiating bone marrow stem cells (from BALB/c or TLR9^-/-^ mice) for 7 days with L-cell-supplemented culture medium (59% Iscove's modified Dulbecco's medium (IMDM), 30% L-929 cell supernatant, 10% FBS, and 1%Pen-Strep mixture). Primary alveolar epithelial cells were isolated using a procedure previously described [34]. Isolated epithelial cells were cultured on fibronectin coated plates for 2 days prior to infection with MOI = 0.01 PFU H1N1 for 24 h prior to harvest of cells for RNA. All cells were incubated in 37°C in 5%CO_2_ until used for experiments.

### Flow cytometry antibodies

TLR9 (J15A7; BD Biosciences; San Jose, CA), IgG1 (MOPC-21; BD Biosciences; San Jose, CA), CD45 (30-F11; BD Biosciences; San Jose, CA), CD11b (M1/70; BD Biosciences; San Jose, CA), CD11c (N418; Biolegend; San Diego, CA), Siglec F (E50-2440; BD Biosciences; San Jose, CA), MHC II (I-A/I-E) (M5/114.15.2; BD Biosciences; San Jose, CA), CD64 (X54-5/7.1; Biolegend; San Diego, CA), F4/80 (BM8 eBiosciences; SanDiego, CA), LY6G (1A8; BD Biosciences; San Jose, CA), CD3 (17A2, BD Biosciences; San Jose, CA), CD90.2 (53–2.1; BD Biosciences; San Jose, CA), CD4 (GK1.5; Biolegend; San Diego, CA) CD8 (53–6.7; BD Biosciences; San Jose, CA), NKP46 (29A1.4; Biolegend; San Diego, CA), CD19 (1D3; BD Biosciences; San Jose, CA), Fc Block(CD16/CD32) (2.4G2; BD Biosciences; San Jose, CA). The NP antibody used was MA1-7322 from Thermofisher (Waltham, MA) conjugated to FITC.

### Model of infection

Influenza infections were done with intranasal instillation of 20μl of PBS containing 100 plaque forming units (PFUs) of PR8 to mice that were anesthetized with a mixture of ketamine and xylazine. Mock infections were PBS alone. MRSA infections were done intratracheally and the dose was always intended to be 7x10^7^CFUs per mouse but instillations through all the experiments came in the range of 5x10^7^CFUs-2x10^8^CFUs.

### Survival assays

Mice were infected intranasally with 100 PFUs of PR8 for 5 days before intratracheal instillation of 7x10^7^ CFUs of MRSA. All mice were anesthetized with a mixture of ketamine and xylazine before viral or bacterial infection. Mice were monitored daily during the course of infection and weighed each morning. Mice were euthanized when reaching a weight loss of greater than 25%. In supplemental data, a survival assay was carried out to MRSA alone as well.

### Plaque assay and viral M1 expression

Madin-Darby canine kidney (MDCK) cells obtained from Dr. Adam Lauring (University of Michigan) were used for the viral titer quantification from whole lungs of infected mice. Briefly, 2x10^5^ MDCK cells were grown in 12-well plate until a well-covered cell monolayer was achieved. Cells were then incubated with serial dilutions of homogenized lungs from infected mice. Cells were washed in 1x MEM-BSA (DMEM, L-Glutamine, amphotericin, Pen-strep, and 10% BSA) medium, then incubated with gentle agitation for an hour at 37°C with virus containing sample prior to the addition of a MEM-BSA & 3% Carboxy Methyl Cellulose overlay containing 2.5 mg/mL trypsin (within phenol red). Plates were left at 37°C for 48–72 hours before adding a crystal violet solution for plaque quantification. In some experiments viral gene expression was determined by levels of H1N1 M1 gene expression by real-time PCR.

### IFN-γ neutralization

PR8 infected mice were treated with 200μg of IFN-γ neutralizing antibody (XMG1.2; BioXcell; West Lebanon, NH) or its isotype control (HRPN; BioXcell; West Lebanon, NH) intraperitoneally on day 5 post-PR8 infection prior to a secondary bacterial infection with MRSA (7x10^7^ CFUs). Successful neutralization was confirmed by measuring IFN-γ levels in broncholalveolar lavage fluid (BALF) by ELISA and by verifying that bronchoalveolar lavage fluid from neutralized mice was unable to activate an IRF-1 reporter cell line. Isotype-treated mouse BALF activated the fluorescent IRF reporter at a level of 1 ±0.1 fold, while anti-IFNγ treated BALF showed 5-fold lower activation at 0.21 ±0.03 (P = 0.0007).

### Tetrazolium dye reduction assay of bacterial killing

2x10^5^ alveolar macrophages (AMs) or BMDMs per well were seeded into duplicate 96 well plates: one control and one experimental plate. Cells from both plates were treated with IgG-opsonized bacteria (MOI 50:1) for 30 minutes at 37°C. The cells on the experimental plate were washed twice with PBS and then incubated with or without IFN-γ (10ng/ml) at 37°C for 120 minutes, whereas the control plate was keep in 4°C with 0.5% saponin in growth medium. After 120 minutes, 0.5% saponin in growth medium was added to experimental plate and Thiazolyl blue Tetrazolium Bromide assay was performed for each plate as explained in [35]. Opsonized phagocytosis values were obtained from control plates.

### Non-opsonized phagocytosis assay

Heat inactivated and fluorescein isothiocyanate (FITC)-labeled bacteria was added into a half-area black 96 well plate containing 2x10^5^ cells per well at a MOI of 1:300 Cells were allowed to ingest bacteria for 120 minutes before trypan blue was added to quench extracellular fluorescence. Intracellular fluorescence was obtained by measuring fluorescence at 485ex/535em using a Spectra M3 microplate reader.

### Enzyme-linked immunosorbent assay (ELISA) and albumin quantification

Cytokine measurement was performed with the use of R&D duo set ELISA kits for murine IFN-γ, TNF-α, IL-1β, IL-6, IL-10, and IL-17. Murine albumin measurement was performed with the use of the Bethyl Laboratory (Montgomery, TX) albumin ELISA kit.

### Immunoblotting

TLR9 immunoblotting was performed in total lung immune cells after collagenase digestion. In these experiments, cell lysates were obtained using RIPA buffer with protease inhibitor. Briefly, total protein from lung immune cells was separated in a polyacrylamide gel using a mini gel tank (Invitrogen, Carlsbad, CA), following by transferring protein to a polyvinylidene fluoride (PVDF) membrane that was blocked with 5% non-fat milk followed by overnight incubation with a polyclonal anti-TLR9 antibody(PA5-20202; Invitrogen; Carlsbad, CA).

### Quantitative real-time PCR

mRNA was isolated using TRIzol according to the manufacturer’s instructions. Relative gene expression measurements were achieved with the use of a Step-one plus real-time PCR system from Applied Biosystems (Foster City, CA). Gene-Specific primers and probes were designed with the GenScript Real-time PCR primer design software (Genscript Biotech Corporation, Piscataway, NJ). Table 1 shows the sequence of primers and probes used in the current studies.

**Table 1:** Primers and probes used throughout experiments.

Name	Sequence (5’-3’)	Strand	Modification
TLR2	ATGGGCTCGGCGATTTC	Forward	
	ATGCAACCTCCGGATAGTGACT	Reverse	
	CGGAGTCAGACGTAGTGAGCGAG	Probe	5’Fam-3’Tamra
TLR3	GCCCTCCTCTTGAACAACGC	Forward	
	ACTTCAGCCCAGAGAAAGTGCT	Reverse	
	ACCAGCTGCTGGCCACCAGCGAG	Probe	5’Fam-3’Tamra
TLR4	AAGGAGTGCCCCGGTTTC	Forward	
	CACAATAACCTTCCGGCTCTTG	Reverse	
	TGCCAACATCATCCAGGAAGGCT	Probe	5’Fam-3’Tamra
TLR7	TCTGCAGGACCTCTGTCCTTG	Forward	
	TGATTGTCTGTGGTCAGGGCAT	Reverse	
	TGGCCTGCAAATCCACAGGCTCACCCA	Probe	5’Fam-3’Tamra
TLR9	GAGTACTTGATGTGGGTGGGAAATT	Forward	
	GCCACATTCTATACAGGGATTGG	Reverse	
	CCGTCGCTGCGACCATGCC	Probe	5’Fam-3’Tamra
INOS	ACATCAGGTCGGCCATCACT	Forward	
	CGTACCGGATGAGGCTGTGAAT	Reverse	
	CCCCACCGGAGTGACGGCA	Probe	5’Fam-3’Tamra
MARCO	CCTGGACGAGTCGGTCAGAA	Forward	
	CTTCAGCTCGGCCTCTGTT	Reverse	
	CCACGCGTCCGGATCATGGGT	Probe	5’Fam-3’Tamra
SRAI/II	TGAAGGACTGGGAACACTCACA	Forward	
	CAGTAAGCCCTCTGTCTCCCTTT	Reverse	
	TTCATTCAAGGGCCTCCTGGACCC	Probe	5’Fam-3’Tamra
Influenza A virus	GGACTGCAGCGTAGACGCTT	Forward	
	CATCCTGTTGTATATGAGGCCCAT	Reverse	
	CTCAGTTATTCTGCTGGTGCACTTGCCA	Probe	5’Fam-3’Tamra
β-Actin	CCGTGAAAAGATGACCCAGATC	Forward	
	CACAGCCTGGATGGCTACGT	Reverse	
	TTTGAGACCTTCAACACCCCA	Probe	5’Fam-3’Tamra

### Statistical analysis

Graphpad Prism version 7 software (Graphpad Prism Software Inc., La Jolla, CA) was used to analyze experimental results. When groups of two were compared, student’s T-test was used to determine statistical significance. Groups of ≥ 3 were compared using one-way analysis of variance with Bonferroni multiple mean comparisons.

## Supporting information

S1 Fig TLR9-/- and Balb/c mice show equivalent susceptibility to MRSA alone. Balb/c and TLR9-/- mice were (A) weighed on day 0 to verify there was no difference in their starting weight. Mice of both genotypes were infected with 1.3 x 108 CFU MRSA via oropharyngeal aspiration. (B) Mice were weighed daily and (C) assessed for survival through day 7. TLR9-/- mice lost more weight on day 3 post-infection, but recovered by day 7. **P<0.01 by Student’s t-test at day 3.
(TIF)

S2 Fig Reduced MRSA clearance, and exacerbated lung tissue injury in the lung post-IAV infection. Bacterial load measurement in the whole lung of mice infected, or not, with (A) 10 PFUs or (B) 100 PFUs and co-infected with MRSA 5, 7 or 10 days post-H1N1 infection. Albumin measurements from the BALF of (C) 10 PFUs or (D) 100PFUs IAV-infected or not mice for 5, 7 and 10 days and co-infected with MRSA for 24 hours. Relative expression of M1 viral gene in lungs of mice infected with 10 PFUs (E) or 100 PFUs (F) of IAV, samples were taken on days 3, 5, 7, and 10 post-infection. Statistics are ANOVA with Tukey’s post-test. *P<0.05,**P<0.01, ***P<0.001, ****P<0.0001; # two mice died in this group before bacterial load measurement. 0 dpi mice were infected with placebo, PBS, 5 days before MRSA coinfection.
(TIF)

S3 Fig Flow gating strategy for Fig 6B in the main text. Gates are shown for one representative sample of each genotype of mice dual infected with H1N1 and MRSA on day 5. Balb/c shown in top panels and TLR9^-/-^ mouse shown on bottom.
(TIF)

S4 Fig Immune cell profiles in TLR9-/- mice post-IAV infection. Absolute number of lung immune cells post-lung collagenase digestion in BALB/c and TLR9^-/-^ mice that were infected with IAV (100 PFUs, H1N1) for 5 days. Total lung cells counted by hemocytometer and immune cell quantification was done by flow cytometry; gating was as follows: neutrophils (CD45^+^,CD11b^+^,MHCII^-^,Ly6G^+^); conventional dendritic cells (CD45^+^,CD11c^+^,MHCII^+^,CD64^-^); AMs (CD45^+^,CD11c^+^,Siglec F^+^,CD64^+^); interstitial Macs (CD45^+^,CD11b^+^,MHCII^+^,Siglec F^-^, CD64^+^); B cells (CD45^+^CD90.2^-^CD19^+^); CD4 T cells (CD45^+^CD90.2^+^CD4^+^); CD8 T cells (CD45^+^CD90.2^+^CD4^-^); Th1 (CD45^+^CD90.2^+^CD4^+^,IFN-γ^+^); Th2 (CD45^+^CD90.2^+^CD4^+^IL-4^+^); Th17 (CD45^+^CD90.2^+^CD4^+^IL-17a^+^); Tregs (CD45^+^CD90.2^+^CD4^+^Foxp3^+^). Statistics are student T test between comparative groups; ns = non-significant.
(TIF)

S5 Fig TLR9-/- mice have no difference in clearance of *Streptococcus pnuemoniae* and have no difference in IFN-γ. (A) Lung bacterial burden and (B) cytokine levels in BALB/c and TLR9^-/-^ mice infected with IAV (100 PFUs, H1N1), or treated with PBS, 5 days prior to *Streptococcus pneumoniae* (SPS3) (3x10^5^ CFUs) infection; samples were taken 24 hours post SPS3 infection. Statistics are ANOVA in panel A and student T test between comparative groups in panel B. Non-significant (ns), *P<0.05, **P<0.01, ***P<0.001, ****P<0.0001.
(TIF)
