# Supplementary figures and images for: Influenza-induced immune suppression to methicillin-resistant Staphylococcus aureus is mediated by TLR9
Source: PLoS Pathog. 2019 Jan 25;15(1):e1007560. doi: 10.1371/journal.ppat.1007560 (PMC6364947; doi:10.1371/journal.ppat.1007560)

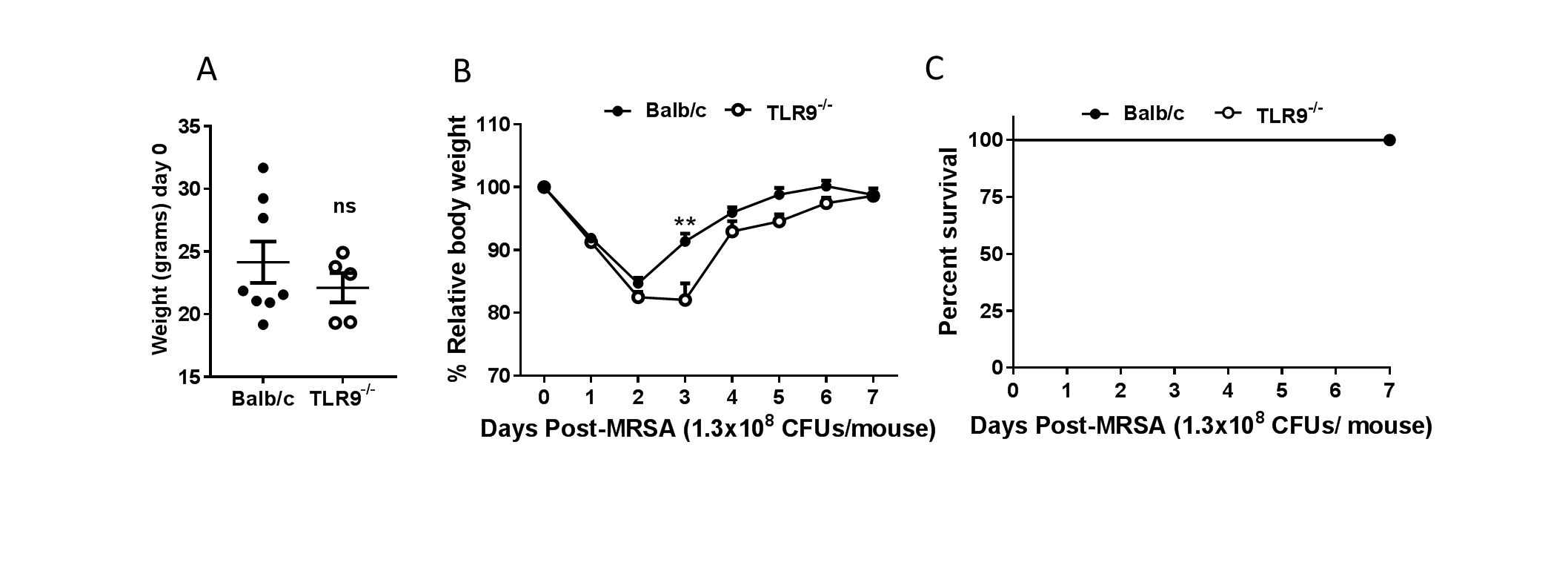

Supplement: S1 Fig — Balb/c and TLR9-/- mice were (A) weighed on day 0 to verify there was no difference in their starting weight. Mice of both genotypes were infected with 1.3 x 10^8^ CFU MRSA via oropharyngeal aspiration. (B) Mice were weighed daily and (C) assessed for survival through day 7. TLR9-/- mice lost more weight on day 3 post-infection, but recovered by day 7. **P<0.01 by Student’s t-test at day 3. (TIF) [file ppat.1007560.s001.tif]

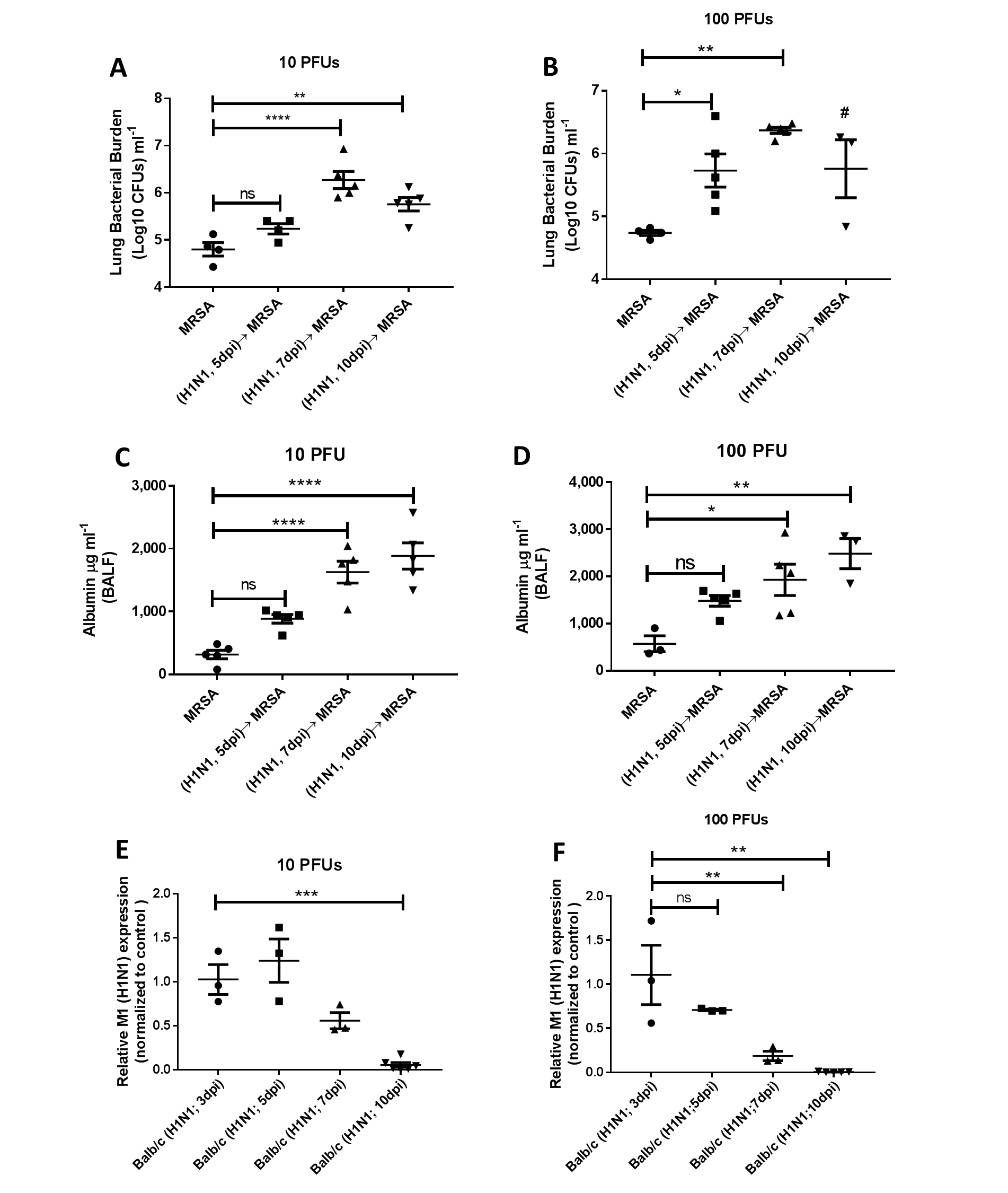

Supplement: S2 Fig — Bacterial load measurement in the whole lung of mice infected, or not, with (A) 10 PFUs or (B) 100 PFUs and co-infected with MRSA 5, 7 or 10 days post-H1N1 infection. Albumin measurements from the BALF of (C) 10 PFUs or (D) 100PFUs IAV-infected or not mice for 5, 7 and 10 days and co-infected with MRSA for 24 hours. Relative expression of M1 viral gene in lungs of mice infected with 10 PFUs (E) or 100 PFUs (F) of IAV, samples were taken on days 3, 5, 7, and 10 post-infection. Statistics are ANOVA with Tukey’s post-test. *P<0.05,**P<0.01, ***P<0.001, ****P<0.0001; # two mice died in this group before bacterial load measurement. 0 dpi mice were infected with placebo, PBS, 5 days before MRSA coinfection. (TIF) [file ppat.1007560.s002.tif]

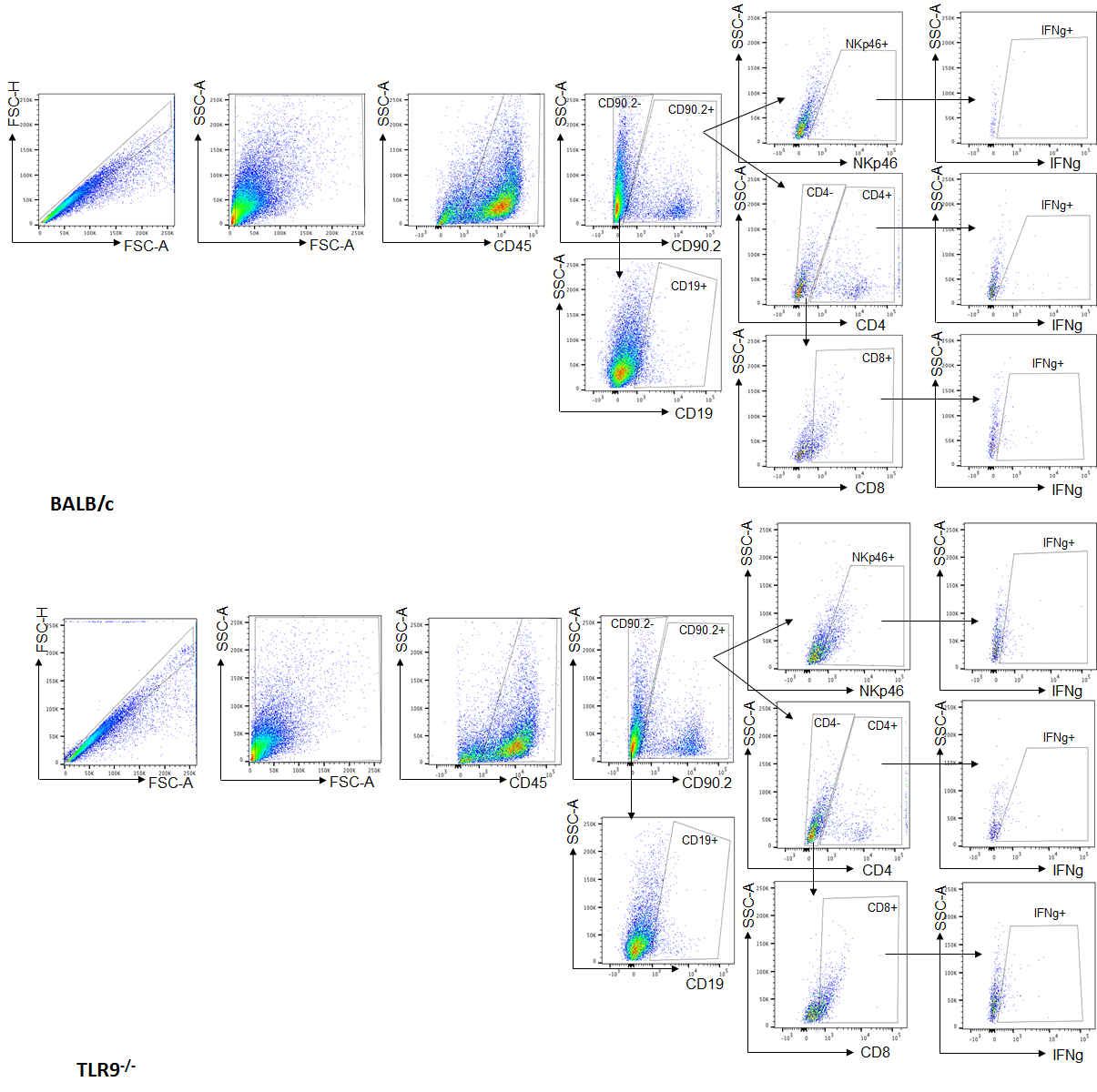

Supplement: S3 Fig — Gates are shown for one representative sample of each genotype of mice dual infected with H1N1 and MRSA on day 5. Balb/c shown in top panels and TLR9-/- mouse shown on bottom. (TIF) [file ppat.1007560.s003.tif]

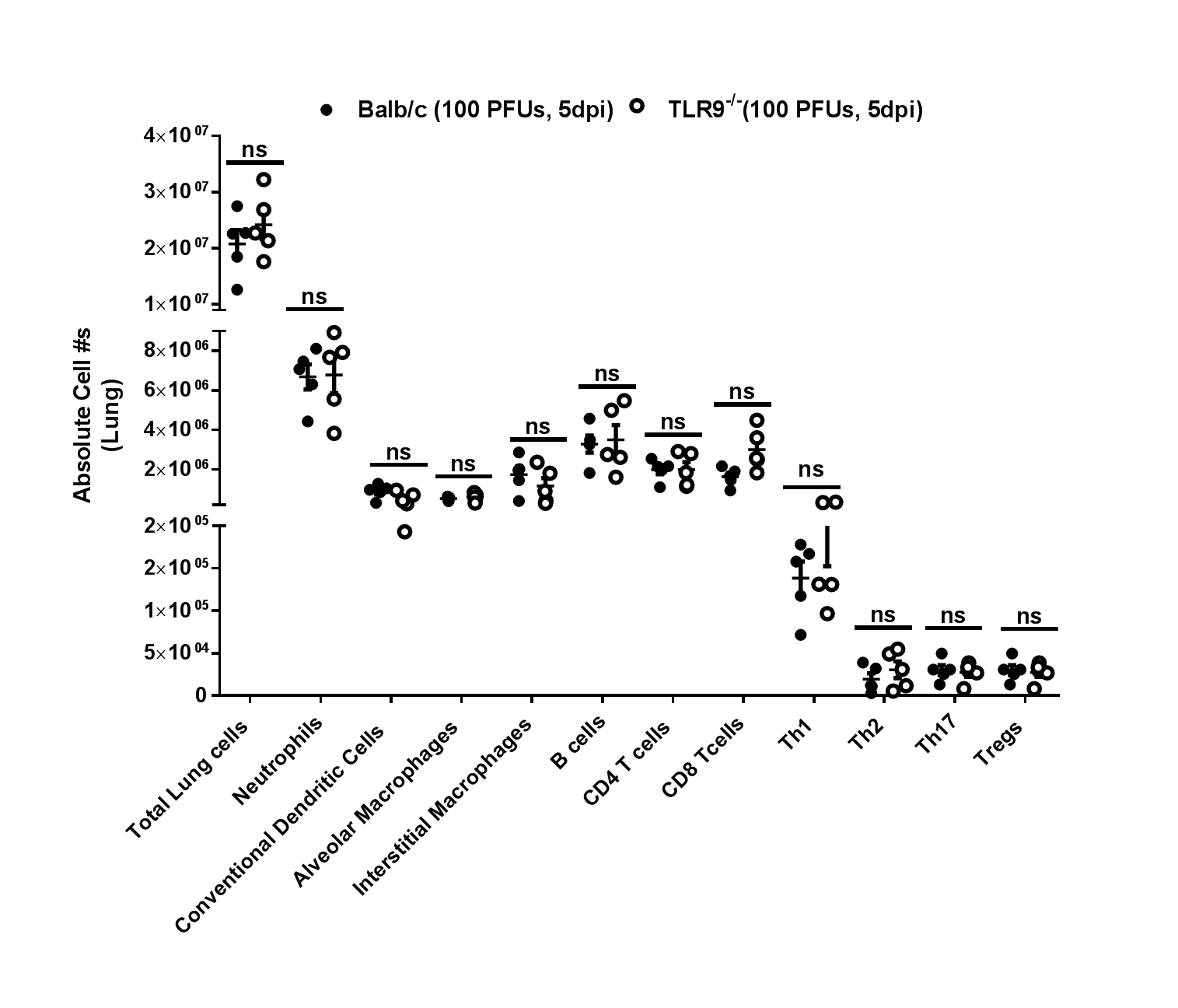

Supplement: S4 Fig — Absolute number of lung immune cells post-lung collagenase digestion in BALB/c and TLR9-/- mice that were infected with IAV (100 PFUs, H1N1) for 5 days. Total lung cells counted by hemocytometer and immune cell quantification was done by flow cytometry; gating was as follows: neutrophils (CD45+,CD11b+,MHCII-,Ly6G+); conventional dendritic cells (CD45+,CD11c+,MHCII+,CD64-); AMs (CD45+,CD11c+,Siglec F+,CD64+); interstitial Macs (CD45+,CD11b+,MHCII+,Siglec F-, CD64+); B cells (CD45+CD90.2-CD19+); CD4 T cells (CD45+CD90.2+CD4+); CD8 T cells (CD45+CD90.2+CD4-); Th1 (CD45+CD90.2+CD4+,IFN-γ+); Th2 (CD45+CD90.2+CD4+IL-4+); Th17 (CD45+CD90.2+CD4+IL-17a+); Tregs (CD45+CD90.2+CD4+Foxp3+). Statistics are student T test between comparative groups; ns = non-significant. (TIF) [file ppat.1007560.s004.tif]

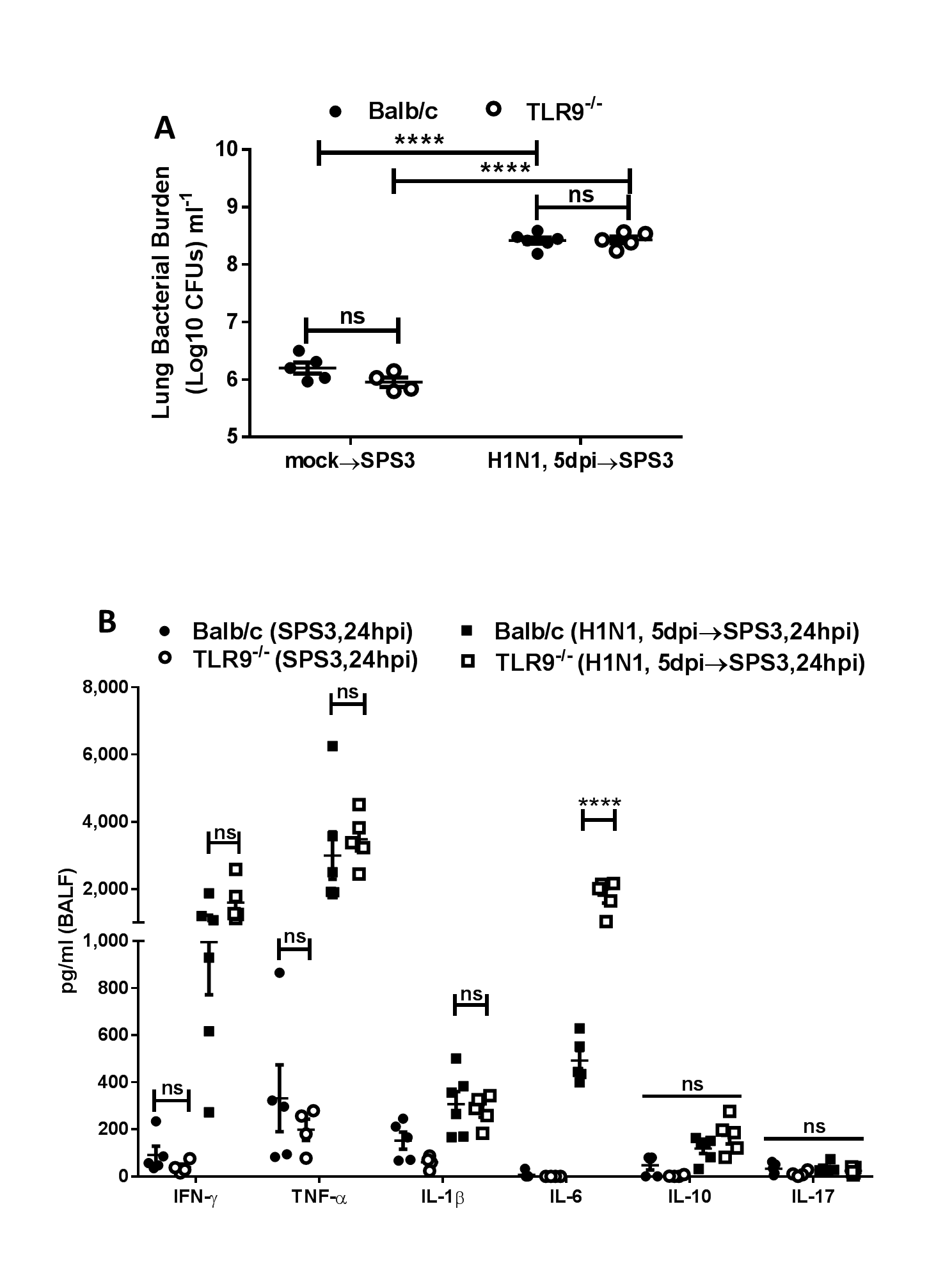

Supplement: S5 Fig — (A) Lung bacterial burden and (B) cytokine levels in BALB/c and TLR9-/- mice infected with IAV (100 PFUs, H1N1), or treated with PBS, 5 days prior to Streptococcus pneumoniae (SPS3) (3x10^5^ CFUs) infection; samples were taken 24 hours post SPS3 infection. Statistics are ANOVA in panel A and student T test between comparative groups in panel B. Non-significant (ns), *P<0.05, **P<0.01, ***P<0.001, ****P<0.0001. (TIF) [file ppat.1007560.s005.tif]
